# Post‐Filler Complications: Beware of Early Warning Signs of Systemic Diseases

**DOI:** 10.1111/jocd.71036

**Published:** 2026-07-01

**Authors:** Hang Wang, Xingcheng Pan, Ziyao Wang, Chihchieh Lo, He Qiu

**Affiliations:** ^1^ State Key Laboratory of Oral Diseases & National Center for Stomatology &National Clinical Research Center for Oral Diseases, Department of Maxillofacial Plastic, Aesthetic and Trauma Surgery West China Hospital of Stomatology, Sichuan University Chengdu China

To the Editor,

The use of injectable materials for facial aesthetic procedures has increased substantially over the past decades, accompanied by a growing spectrum of procedure‐related complications. Early adverse events, such as vascular occlusion, acute hypersensitivity reactions, and immediate post‐injection erythema or edema, are relatively well recognized and can often be promptly identified and managed due to their acute onset and characteristic clinical presentation. In contrast, delayed complications have gained increasing attention in recent years, including foreign body granulomas, delayed hypersensitivity reactions, filler migration, and chronic inflammatory responses of unclear etiology [1]. These reactions may occur months to years after injection, and in rare cases, even decades later, significantly complicating clinical diagnosis and management. Their heterogeneous and often non‐specific manifestations frequently overlap with infectious or allergic conditions, thereby increasing the risk of misdiagnosis and delayed intervention.

All fillers represent exogenous implanted materials that may elicit varying degrees of host immune response. Particularly, collagen‐stimulating agents and animal‐derived fillers may carry inherent immunogenic potential, which could predispose susceptible individuals to abnormal immune activation. Previous studies have suggested that patients with atopic predisposition or heightened immune reactivity may exhibit exaggerated immune responses following filler injection [2].

An often‐overlooked aspect is that systemic immune‐related disorders may initially manifest or become accentuated at filler‐injected sites. In such cases, local findings, such as erythema, swelling, or nodularity, may closely mimic delayed allergic or infectious complications, thereby confounding early clinical assessment. Fail to recognize this possibility may lead to inappropriate treatment and, in some cases, progression to severe systemic involvement.

Therefore, when encountering unexplained, persistent, or atypical inflammatory reactions in previously injected areas, clinicians should extend the differential diagnosis beyond conventional filler‐related complications and maintain a high index of suspicion for systemic conditions, particularly autoimmune diseases. Herein, we report a case of anti‐melanoma differentiation‐associated gene 5 (MDA5) positive dermatomyositis (MDA5‐DM) presenting initially as localized inflammatory changes in facial filler‐injected regions. This case highlights that injection sites may function not only as loci of local complications but also as potential windows for the early detection of systemic immune disorders, providing important implications for clinical decision‐making and patient safety.

## 1 Case Presentation

**FIGURE 1:**
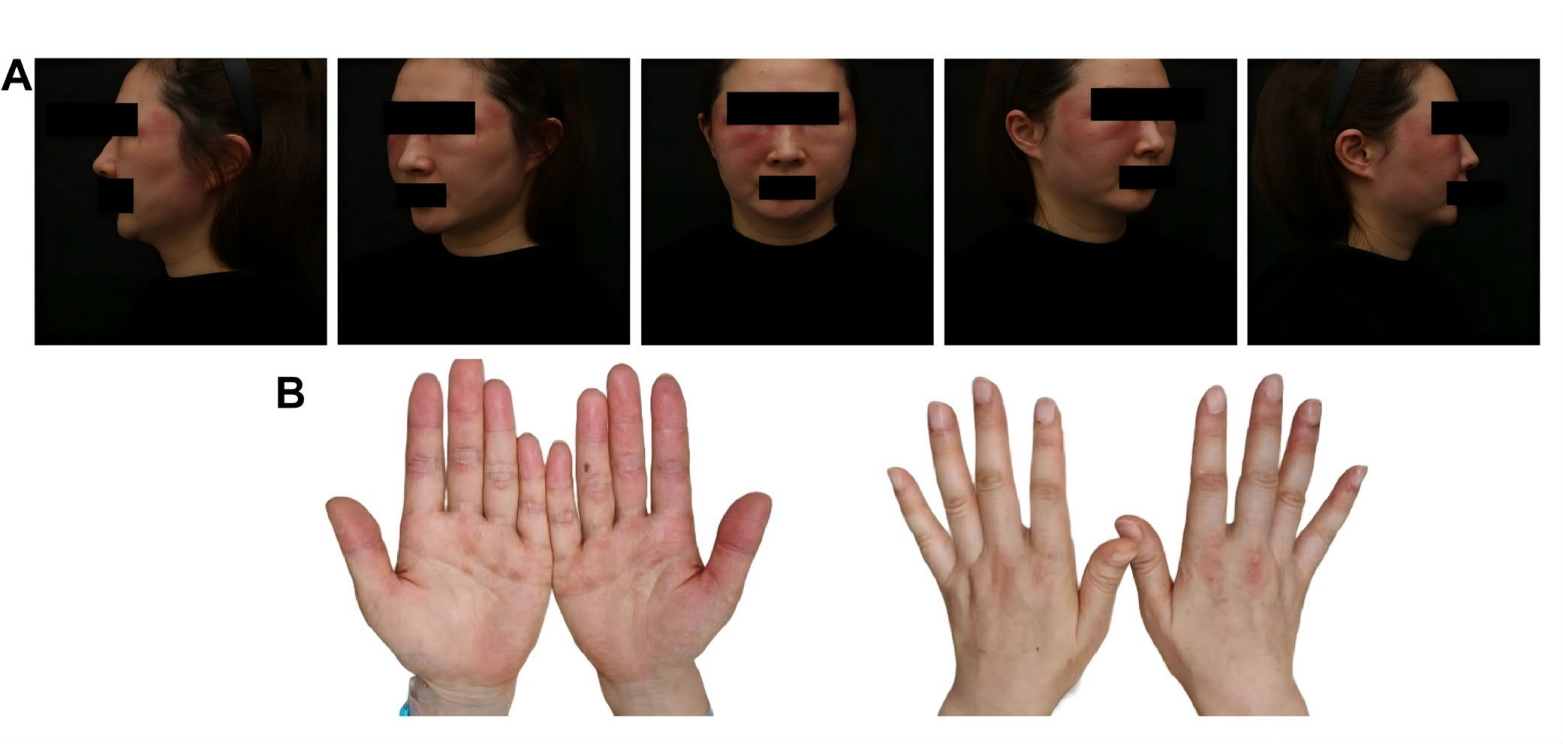
Facial erythema and edema (A) accompanied by erythematous papules on the joints of both hands (B).

Written informed consent was obtained from the patient, and this study was conducted according to the Declaration of Helsinki guidelines. A 40‐year‐old woman presented with progressive facial erythema and swelling. She had undergone multiple collagen stimulants injections (ELLANSÉ, Aqtis Medical, Utrecht, the Netherlands) in the bilateral temporal, infraorbital, and cheek regions more than 2 years earlier. One month prior to presentation, she developed spontaneous erythema and swelling in the right temporal region without identifiable triggers, which rapidly progressed to involve the bilateral temporal and infraorbital areas (Figure 1A). She was evaluated by multiple specialties and treated empirically for delayed filler‐related infection or hypersensitivity with systemic anti‐inflammatory therapy, oral corticosteroids, antihistamines, and local interventions, with no clinical improvement.

**FIGURE 2:**
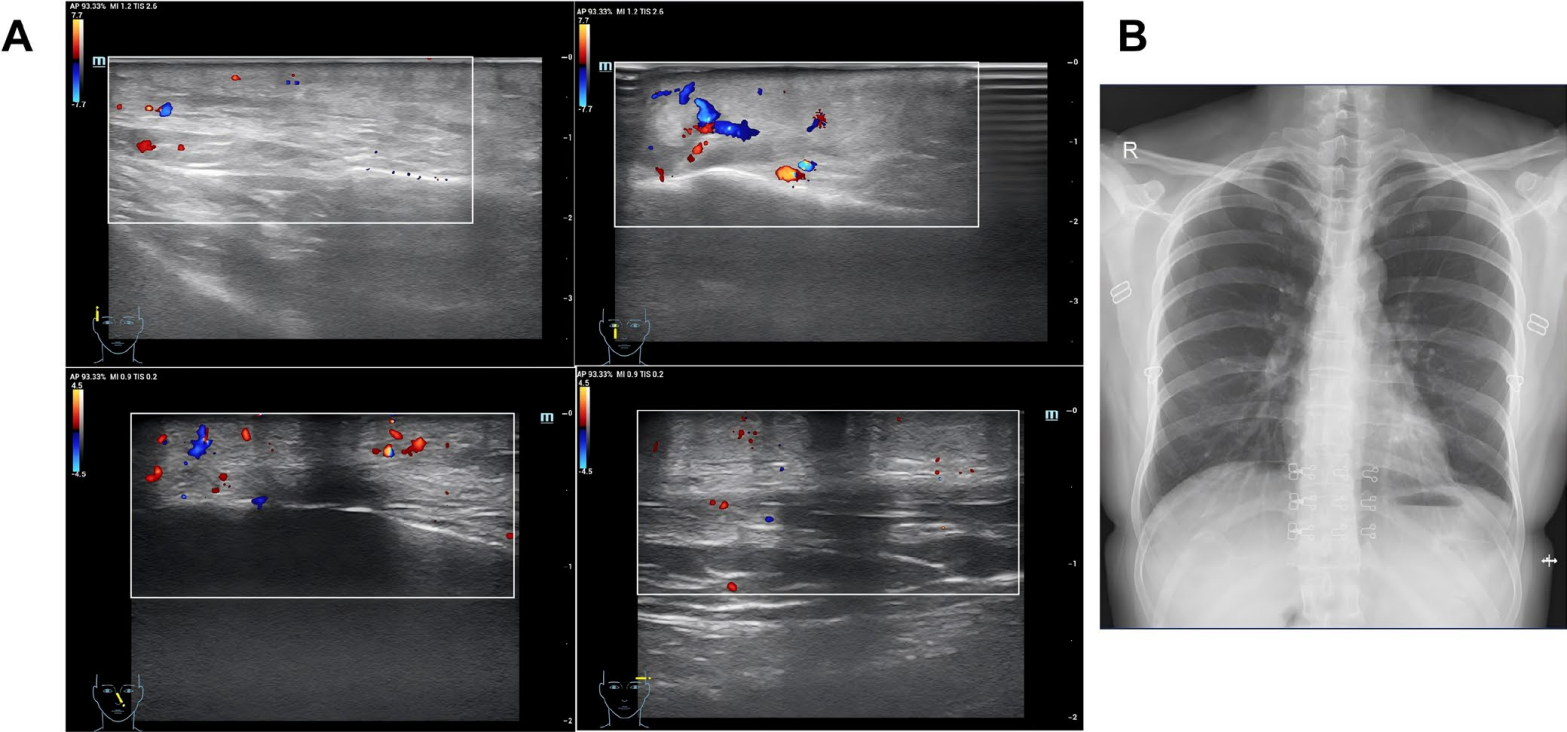
High‐frequency ultrasound images of the swollen facial filler‐injected regions (A) and chest radiography (B).

On examination, extensive erythema and swelling were noted in the bilateral temporal and infraorbital regions, more prominent on the right. Palpation demonstrated firm subcutaneous tissue with increased local skin temperature, without tenderness and fluctuation. In addition, erythematous papules were observed on the palms and interphalangeal joints (Figure 1B). Three days after admission, the patient developed progressively worsening weakness of both upper extremities, accompanied by mild shortness of breath and dyspnea. High‐frequency ultrasound demonstrated diffuse subcutaneous thickening with increased echogenicity and multiple ill‐defined hypoechoic lesions showing posterior acoustic attenuation, suggestive of an inflammatory process (Figure 2A). Laboratory investigations, including serum biochemical analysis, infectious disease markers, urinalysis, electrocardiography, and chest radiography (Figure 2B), revealed no significant abnormalities. Following multidisciplinary consultation with dermatology and rheumatology, immunological testing revealed abnormalities in CD3 lymphocyte subsets, including significantly reduced absolute counts of CD3, CD4, B, and NK cells. Myositis‐specific antibody testing demonstrated a markedly elevated anti–MDA5 antibody level (IgG: 608.62 U/mL). The patient was subsequently transferred to the rheumatology department, where the diagnosis of anti‐MDA5‐positive dermatomyositis (MDA5‐DM) was further confirmed.

## 2 Discussion

Delayed inflammatory reactions following dermal filler injections are increasingly recognized in aesthetic practice and are commonly attributed to foreign body responses, biofilm‐associated infection, or delayed hypersensitivity. However, as demonstrated in the present case, such localized inflammatory manifestations may occasionally represent the initial presentation of an underlying systemic disorder rather than a purely filler‐related complication. Fillers may act as immune triggers, activating host responses through both innate and adaptive pathways. So the filler‐injected sites may become localized foci of amplified immune activation.

MDA5‐DM is a severe autoimmune disease characterized by the presence of anti‐MDA5 antibodies, cutaneous vasculopathic manifestations, and a high incidence of rapidly progressive interstitial lung disease (RP‐ILD) [3]. This subtype often presents with minimal or absent muscle weakness but carries a high mortality risk due to pulmonary involvement. In this case, the initial manifestations were confined to previously injected facial regions and preceded systemic disease. The early presentation closely mimicked filler‐related inflammatory or allergic reactions, leading to repeated misdiagnosis. The subsequent development of violaceous papules over the joints, together with the detection of anti‐MDA5 antibodies, was critical in establishing the diagnosis of MDA5‐DM. This case highlights that filler‐injected sites may act as sentinel locations, where systemic immune dysregulation becomes clinically apparent at an early stage. Therefore, persistent, progressive, or atypical reactions should raise suspicion for underlying systemic disease and warrant multidisciplinary evaluation.

A plausible explanation for this phenomenon is that injected regions, characterized by altered extracellular matrix architecture, local microtrauma, and the presence of foreign material, may act as immune amplification niches. These microenvironments may enhance local innate immune activation, including macrophage and dendritic cell signaling, thereby amplifying interferon‐mediated pathways central to MDA5‐DM pathogenesis [4]. As a result, systemic immune activation may preferentially manifest in these sites before becoming generalized.

Delayed post‐filler inflammatory reactions are often managed empirically with anti‐infective therapy, anti‐allergic treatment, or surgical intervention. While such an approach is appropriate in many cases, failure to respond to standard treatments should prompt reconsideration of the diagnosis. Previous studies have also reported that autoimmune diseases, including dermatomyositis and other connective tissue disorders, may mimic or coexist with filler‐related complications [5]. Therefore, persistent, progressive, or atypical reactions should raise suspicion for underlying systemic disease and warrant multidisciplinary evaluation.

In summary, this case expands the current understanding of post‐filler complications by emphasizing the intersection between aesthetic procedures and systemic immunology. It underscores the need for a broader diagnostic perspective and highlights that injection sites may function not only as locations of local adverse events but also as windows into underlying systemic disease. Recognizing this possibility is essential for timely diagnosis, appropriate referral, and prevention of potentially severe systemic complications.

## Author Contributions

**Hang Wang:** conceptualization, project administration, data curation, formal analysis, investigation, writing – original draft. **Xingcheng Pan:** investigation, data curation. **Ziyao Wang:** formal analysis. **Chihchieh Lo:** formal analysis, methodology. **He Qiu:** conceptualization, data curation, formal analysis, investigation, writing – original draft.

## Funding

The authors have nothing to report.

## Ethics Statement

The authors have nothing to report.

## Conflicts of Interest

The authors declare no conflicts of interest.

## Data Availability

The data that support the findings of this study are available from the corresponding author upon reasonable request.
